# Gastrointestinal Quality-of-Life Trajectories after Radiotherapy for Prostate Cancer—Which Patients Suffer from Persisting Problems?

**DOI:** 10.3390/cancers15174295

**Published:** 2023-08-28

**Authors:** Michael Pinkawa

**Affiliations:** Department of Radiation Oncology, MediClin Robert Janker Klinik, 52074 Bonn, Germany; michael.pinkawa@mediclin.de; Tel.: +49-228-5306-4100

Gastrointestinal toxicity, particularly in relation to rectal bleeding, is regarded as the dose-limiting toxicity in radiotherapy for prostate cancer, and it is the most frequent focus of toxicity studies [1]. In the last two decades, patient-reported quality-of-life data has gained increasing relevance and are commonly considered in all currently published prospective studies that evaluate new radiotherapy concepts. A prospective quality-of-life study always includes a baseline score and changes in this score over time [2]. For radiotherapy, we differentiate an acute phase of toxicity, including the time during the actual treatment and three months after the treatment, from a chronic phase of toxicity, including long-term problems. Chronic toxicity can persist or even progress, thereby constantly impairing patients’ quality of life [3]. 

Noale et al. [4] differentiated between different gastrointestinal quality-of-life trajectories, aiming to identify patients with persisting gastrointestinal problems. This large prospective Italian study enrolled 1705 patients who responded to the University of California Los Angeles—Prostate Cancer Index (UCLA-PCI) questionnaire, which was developed before the introduction of the Expanded Prostate Cancer Index (EPIC) questionnaire, which was a modified PCI questionnaire [5]. The EPIC questionnaire is now more commonly used. This analysis focused on a period of up to 24 months after radiotherapy. Patients were recruited over a period of one year. 

Commonly, quality of life is reported based on group means. Growth mixture models, as presented in the study by Noale et al. [4], are used for the post hoc identification of multiple subpopulations, describing longitudinal changes within each sub-population. Three different trajectories considering bowel scores have been differentiated:

Trajectory 1 (10% of patients)—low baseline scores with acute decline and only limited recovery.
Trajectory 2 (23% of patients)—low baseline scores without acute and long-term decline.
Trajectory 3 (71% of patients)—high baseline scores with gradual decline.

The cited authors have identified the use of older treatment techniques and patient comorbidities as significant risk factors for worse trajectories. In particular, and corresponding with prior studies, diabetes was reported to be a risk factor for severe bowel problems [6]. Current treatment techniques include intensity-modulated and image-guided radiotherapy (IMRT and IGRT), which are used to deliver a treatment with the best conformity (a high dose primarily focused on the prostate) and accuracy (localizing the prostate before each treatment fraction, thus ensuring that the dose is actually reaching the prostate) [7,8]. With an IMRT technique, the dose administered to the rectum can be decreased considerably in comparison to the older three-dimensional conformal techniques. IMRT—particularly when applied as volumetric modulated arc therapy (VMAT), wherein both a gantry and a multi-leaf collimator are constantly rotating during the delivery of radiation—is regarded as a standard for prostate cancer radiotherapy. When applying IGRT, smaller safety margins can be used, once again decreasing the dose administered to the rectum. Both techniques (IMRT and IGRT) are important for improving tumor control and decreasing treatment-associated toxicity. They allow for a safe dose escalation to improve tumor control without considerably increasing treatment-related toxicity [9].

According to current recommendations, to account for inter-fractional prostate movement, daily IGRT should preferably be used for conventionally fractionated radiotherapy but is explicitly recommended for hypo-fractionated radiotherapy. The minimum standard must be based on either fiducial markers or CT-based approaches with soft-tissue matching. A combination of fiducial markers with soft-tissue matching is preferred. With higher single fractions, i.e., >5 Gy, frequently described as extreme hypofractionation, the ideal tracking of intrafraction motion may be considered [7]. 

A modern technique for reducing the dose administered to the rectum with increasing importance in prostate cancer radiotherapy is the application of rectal spacers that are placed between the prostate and rectal wall. The injection of a spacer results in a mean prostate–rectum distance of about 1 cm [10]. Experience with this technique and the number of published studies, including prospective randomized studies, are increasing [11]. The greatest body of experience is available for the use of hydrogel spacers [2], but a large randomized study has also recently been published for a hyaluronic acid spacer [12]. Hydrogel spacers have been used for all available external beam fractionation concepts and brachytherapy [10,11,13]. Studies have shown that spacers can effectively prevent rectal toxicity and a trajectory with gradual gastrointestinal quality-of-life decline over several years [2]. 

For the interpretation of the results in this field, it is important to understand the different forms of radiobiological development of chronic toxicities. Chronic toxicity can be independent from acute toxicity; thus, toxicity can develop over years, even without presenting acute problems. However, consequential late effects play an important role in the development of chronic rectal toxicity [3]. Acute effects can persist if physical or mechanical stress prevents healing. Diabetes can prevent healing if blood perfusion in a particular area is impaired. The interaction between pro-inflammatory cytokines through oxidative stress mechanisms, induced by ionizing radiation, has also been proposed as an explanation for higher rectal toxicities in diabetic patients [14]. The presence of comorbidities can predict reduced quality-of-life scores after prostate cancer radiotherapy concomitant with androgen deprivation therapy [4,15]. Instead of the number of comorbidities, prior studies have also correlated the Charlson Comorbidity Index with rectal toxicity [16]. It is reassuring for younger patients without comorbidities—and thus a longer life expectancy—to know that they have a good chance of surviving without chronic rectal toxicities.

The prevention of additional physical and mechanical stress is of considerable importance. Preventive measures include, above all, dietary counselling or laxatives to reduce the frequency, volume, and hard consistency of stools. Invasive procedures and biopsies of the rectal wall must be avoided. Extensive interventions may prevent healing and lead to persistent inflammation or fistula formation. Unfortunately, evidence for the beneficial effects of pharmacological interventions is scarce. Antioxidants (such as retinyn palmitate, i.e., vitamin A) can reduce symptoms of chronic radiation proctitis, such as bleeding and diarrhea [17].

Important limiting factors of the presented results are the limited time period of only 24 months after treatment and decreasing response rates with longer follow-ups (90% after 12 months and 73% after 24 months), with a possible impact on the trajectories. A stable period in the second year after treatment does not guarantee stability over many years. Regarding chronic radiation-associated toxicity, far longer time periods need to be considered so that studies ensuring a longer follow-up of five and more years after treatment can be supported [18]. To understand rectal toxicity, it is crucial to understand predisposing factors in order to be aware of patients at greater risk and to understand the different trajectories that have been described, particularly the association with acute toxicity but also an independent development of toxicity over many years.
